# A Comprehensive *In Vitro* and *In Silico* Study of Acteoside: Cytotoxicity, Molecular Interactions, and DFT Reactivity Profiles

**DOI:** 10.1021/acsomega.6c04617

**Published:** 2026-10-26

**Authors:** Erdi Can Aytar, Metin Çalışkan, Emine İncilay Torunoğlu

**Affiliations:** † Uşak University, Faculty of Agriculture, Department of Horticulture, Uşak 64200, Türkiye; ‡ Uşak University, Faculty of Medicine, Department of Medical Biology, Uşak 64200, Türkiye; § Necmettin Erbakan University, Faculty of Medicine, Department of Medical Biochemistry, Konya 42080, Türkiye

## Abstract

In this study, the cytotoxic effects of acteoside were comparatively evaluated in four cancer cell lines (MiaPaCa-2, HL-60, A549, and MDA-MB-231). Based on the greater sensitivity observed in MiaPaCa-2 cells, molecular docking against the EGFR/HER2 tyrosine kinase domain was subsequently performed to investigate a potential molecular interaction relevant to the observed cytotoxic response in all four cell lines, acteoside reduced cell viability in a dose-dependent manner, with the lowest IC_50_ observed in MiaPaCa-2 cells (IC_50_ = 166.9 μM). Molecular docking analysis revealed a favorable binding affinity of acteoside toward the EGFR/HER2 tyrosine kinase domain, with a predicted binding energy of −8.9 kcal/mol. DFT calculations revealed solvent-dependent electronic properties, while frontier molecular orbital, molecular electrostatic potential (MEP), noncovalent interaction, and electron localization analyses identified electron-rich reactive regions and potential interaction sites within the acteoside molecule. Overall, acteoside exhibits varying levels of cytotoxic activity across the tested cell lines, and further studies are required to elucidate its mechanism of action.

## Graphical abstract

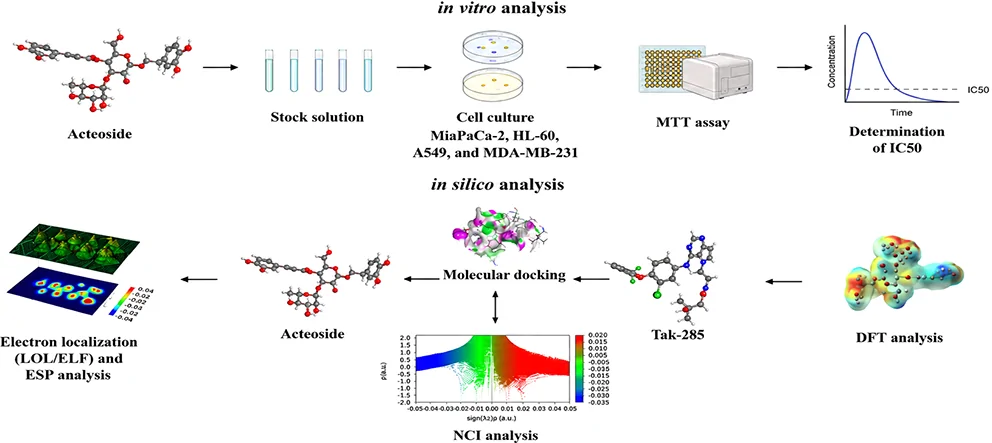

## Introduction

Cancer cells often enhance the production of metabolic components required for protein, lipid, and nucleic acid synthesis to sustain rapid proliferation, supporting their survival and growth.^1^ Globally, cancer poses a growing threat to human health, being one of the leading causes of death worldwide. According to the Global Cancer Observatory (GLOBOCAN) 2020, approximately 10 million cancer-related deaths and 19.3 million new cases were reported worldwide in 2020, and global cancer incidence is expected to reach 28.4 million by 2040.^2^ Pancreatic cancer is among the most lethal malignancies, with poor prognosis and therapeutic resistance representing significant barriers to patient survival.^3^ Similarly, acute myeloid leukemia is a heterogeneous group of diseases characterized by clonal expansion and impaired differentiation of myeloid progenitor cells in the peripheral blood and bone marrow.^4^ Even in patients achieving remission with chemotherapy, relapses occur in approximately two-thirds of cases due to chemoresistance and the persistence of leukemic cells below morphologic detection.^5^

Lung cancer is also a prevalent malignancy with high morbidity and mortality, with an estimated 10,000 new cases annually, largely associated with risk factors such as tobacco use, physical inactivity, and modern dietary habits.^6^ Although surgical, chemotherapeutic, immunotherapeutic, and radiotherapeutic approaches remain standard treatments, their efficacy is often limited by off-target effects, toxicity to normal cells, and the development of drug resistance.^7^ Consequently, the development of novel, cost-effective, and biocompatible therapies targeting cancer cell proliferation has become a critical priority.^8^

Breast cancer represents one of the most common malignancies in women, accounting for approximately 7–10% of all cancers. In 2016, 246,660 invasive breast cancer cases were reported in the United States, with approximately one-sixth resulting in mortality. Triple-negative breast cancer (TNBC), characterized by the absence of estrogen receptor (ER), progesterone receptor (PgR), and human epidermal growth factor receptor type 2 (HER2) expression, is particularly aggressive and unresponsive to hormone or anti-HER2 therapies.^9^ Triple-negative breast cancer (TNBC) exhibits a high recurrence rate, strong invasiveness, and typically affects younger and obese women, emphasizing the need for effective treatment strategies.

Active compounds, particularly phenolic compounds, exhibit therapeutic properties owing to their antiproliferative, anti-inflammatory, and antioxidant activities.^10^ Acteoside is a caffeoyl phenylethanoid glycoside isolated from various medicinal plants, including *Verbascum phlomoides*,^11^ *Buddleja globosa*,^12^ and *Plantago australis*.^13^ This compound exhibits a wide range of biological activities, including antimicrobial,^12^ anti-inflammatory,^14^ anticancer,^15^ antioxidant, cytoprotective,^16^ and neuroprotective effects.^17^ Acteoside exhibits cell- and context-dependent biological activity. In addition to its anticancer properties, acteoside has been shown to modulate immune responses, including the promotion of IL-10 production in human and murine B cells through the TLR4/PI3K signaling axis.^18^ Its antiproliferative and cytotoxic effects also vary considerably among cancer cell types and experimental conditions.^19,20^ These context-dependent responses may be associated with the multiple biological processes modulated by acteoside, including inflammatory and oxidative-stress signaling, mitochondrial regulation, cell-cycle control, and apoptosis.^21–24^ In particular, recent studies have demonstrated dose- and time-dependent cytotoxic effects of acteoside in several cancer cell lines, including MCF-7 and MDA-MB-231 breast cancer cells,^19^ while significant cytotoxicity and inhibition of proliferation and migration have also been reported in MCF-7 cells.^20^ These findings indicate that the biological response to acteoside may vary depending on the cellular context, highlighting the need to comparatively investigate its effects across different cancer model. Accordingly, cancer cell lines representing different tissue origins and biological characteristics were selected, with priority given to models of cancer types associated with high incidences or mortality rates. Within this framework, the present study aimed to comparatively evaluate the cytotoxic effects of acteoside using pancreatic cancer (MiaPaCa-2), acute promyelocytic leukemia (HL-60), lung adenocarcinoma (A549), and breast cancer (MDA-MB-231) cell models.

## Materials and Methods

### Acteoside Stock Solution and Dilutions

One hundred milligrams of acteoside, with a molecular weight of 624.59 g/mol, was dissolved in 1.563 mL of dimethyl sulfoxide (DMSO) to prepare an approximately 102.4 mM stock solution to achieve the targeted stock concentration. This prepared stock solution was diluted 1:50 with phosphate-buffered saline (PBS) to obtain a 2048 μM solution for the initial concentration required for the study. Starting from this solution, serial 1:1 dilution was prepared using culture media appropriate for each cell line, resulting in acteoside solutions of 1024, 512, 256, 128, 64, and 32 μM, respectively.

### Cell Culture

The cell lines used in this study were human pancreatic adenocarcinoma (MiaPaCa-2), human promyelocytic leukemia (HL-60), human lung adenocarcinoma (A549), and human triple-negative breast cancer (MDA-MB-231) cell lines. MiaPaCa-2 and A549 cells were cultured in Dulbecco’s Modified Eagle Medium (DMEM; Gibco), whereas MDA-MB-231 and HL-60 cells were cultured in RPMI-1640 medium (Gibco). All culture media were supplemented with 10% fetal bovine serum (FBS) and 1% penicillin–streptomycin. Adherent cells (MiaPaCa-2, A549, and MDA-MB-231) were detached using trypsin–ethylenediaminetetraacetic acid (trypsin–EDTA) upon reaching approximately 85% confluence, whereas HL-60 cells were maintained in suspension culture and refreshed at regular intervals. All cells were maintained at 37 °C in a humidified atmosphere containing 5% CO_2_.

For cell viability assays, MiaPaCa-2, A549, and MDA-MB-231 cells were seeded into 96-well plates at a density of 1 × 10^4^ cells/well and allowed to attach for 24 h. HL-60 cells were plated as a suspension at the same density. Subsequently, acteoside concentrations prepared in the range of 32–2048 μM were applied to the cells and exposed continuously for 72 h. At the end of the incubation period, cell viability was determined using the MTT method, and all experiments were conducted in three independent replicates (*n* = 3).

### MTT Method and Analysis

Following completion of the incubation periods, the liquid in all wells was removed. Into each well, 10 μL of 5 mg/mL 3-(4,5-dimethylthiazol-2-yl)-2,5-diphenyl tetrazolium bromide (MTT) solution and 90 μL of culture medium were added to make a total volume of 100 μL. The plates were then incubated at 37 °C for 4 h. At the end of the incubation, 150 μL of 99.9% DMSO was added to dissolve the formed crystals, and the plates were incubated for an additional hour. Cell viability was assessed by measuring absorbance values at 570 nm using a Multiskan Sky Microplate Spectrophotometer (Thermo Fisher Scientific). In calculations, percentage values were obtained by expressing the absorbance value of each sample relative to that of the control group.

### Determination of IC_50_ Values and Statistical Analysis

Statistical analyses were performed using GraphPad Prism software. Dose–response curves were fitted using the four-parameter logistic (4PL) model with log_10_-transformed concentration data, and IC_50_ values were calculated from these curves. Two-way ANOVA was applied to evaluate the effects of cell line and concentration on viability. The Extra-Sum-of-Squares *F* test was used to assess whether differences in IC_50_ values between cell lines were statistically significant. In all analyses, *p* < 0.05 was considered statistically significant.

### Molecular Docking Methodology

In this study, the tyrosine kinase domain of the EGFR/HER2 heterodimer was selected as the molecular target, represented by the high-resolution crystal structure deposited in the Protein Data Bank under the accession code 3POZ. This structure contains the kinase domain bound to a clinically relevant dual EGFR/HER2 inhibitor (TAK-285), providing a well-defined model for studying ligand–protein interactions within the ATP-binding cleft.

Molecular docking is a method that enables the prediction of possible binding modes and molecular interaction mechanisms between bioactive compounds and biological targets. Oyeneyin et al.^25^ selected the EGFR kinase domain (PDB ID: 3POZ) for molecular docking studies because it represents a biologically important target closely associated with cell proliferation in MIAPaCa-2 pancreatic cancer cells.^26,27^ In our study, acteoside exhibited the highest cytotoxic activity by showing the lowest IC_50_ value among the tested cancer cell lines in the MIAPaCa-2 pancreatic cancer cells. Therefore, molecular docking analyses were performed using the EGFR kinase domain (PDB ID: 3POZ), which is associated with the MIAPaCa-2 cell line, to investigate the possible molecular mechanisms of action of acteoside. EGFR is known to be overexpressed in pancreatic cancer and to play an important role in tumor cell proliferation, survival, and invasion.^28^ Furthermore, although acteoside has been reported to exhibit anticancer activity in various cancer types, its molecular mechanism of action in pancreatic cancer has not yet been fully elucidated.^29^ Therefore, the EGFR kinase domain was selected as the target protein to investigate the possible interactions of acteoside with this receptor and evaluate the possible molecular basis of the observed cytotoxic activity. This approach aims not to demonstrate that EGFR is the definitive molecular target of acteoside but rather to investigate possible binding interactions and the potential mechanism of action that may explain the observed biological activity.

In addition, the cocrystallized ligand TAK-285 was selected as the reference compound to enable the comparative evaluation of binding affinities. TAK-285 is a dual EGFR/ErbB1 and HER2/ErbB2 kinase inhibitor that binds to and inhibits both receptors, thereby exhibiting antitumor activity in EGFR/HER2-expressing tumors.^30,31^ Since EGFR and HER2 play critical roles in tumor cell proliferation, the well-characterized inhibitory activity of TAK-285 against these receptors constitutes the primary rationale for its selection as the reference compound in the molecular docking analysis.

As the chemical ligands of interest, acteoside and the crystallographic inhibitor CID 11620908 were selected to represent structurally distinct, yet biologically meaningful molecules. Acteoside is a phenylethanoid glycoside known for its multifunctional bioactivities, while CID 11620908 embodies the canonical binding mode of TAK-285 analogues within the EGFR/HER2 ATP-binding site. This combination enables the comparison of a naturally occurring bioactive compound to a well-characterized synthetic inhibitor, facilitating the interpretation of docking scores and interaction profiles. Both ligands were preprocessed to ensure optimal stereochemistry and energetically favorable conformations prior to docking.

Protein preparation was performed using AutoDock Tools 1.5.7.^32^ All crystallographic water molecules, ions, and nonessential heteroatoms were removed from the 3POZ structure. Polar hydrogens were added, and Gasteiger partial charges were assigned to the macromolecule. The cleaned and fully parametrized protein structure was exported in the PDBQT format for use in AutoDock Vina. The minimized ligand structures were imported into AutoDock Tools 1.5.7, where they were prepared in their default neutral protonation states. Polar hydrogen atoms were added, Gasteiger partial charges were assigned, and rotatable bonds were defined to allow for torsional flexibility during docking. The prepared ligands were finally saved in the PDBQT format for compatibility with AutoDock Vina. Ligand structures were prepared by energy minimization and conversion to three-dimensional format. Each ligand was imported into AutoDock Tools, torsional flexibility parameters were assigned, and Gasteiger charges were calculated. The ligands were then saved in the PDBQT format to ensure compatibility with the docking engine.

Docking simulations were performed using AutoDock Vina (version 1.2.0).^33^ The docking grid was defined based on the ATP-binding site coordinates used by Oyeneyin et al.,^25^ with the grid center positioned at *x* = 20.37, *y* = 32.82, *z* = 14.89. The grid dimensions were adjusted to enclose the catalytic core and surrounding hydrophobic pockets of the kinase domain, ensuring full coverage of the functional binding region. The exhaustiveness parameter was set to 8, providing sufficient conformational sampling while maintaining computational efficiency. Following docking, all binding poses were ranked by predicted binding affinity (kcal/mol). The top-scoring conformations were examined for structural compatibility with the binding pocket, and detailed interaction analyses including hydrogen bonding, hydrophobic contacts, and π-stacking interactions were conducted using Discovery Studio Visualizer 2021 to characterize the molecular interaction network within the EGFR/HER2 ATP-binding cavity.

### DFT Computational Analysis

Density functional theory calculations were performed to investigate the molecular geometry, frontier-orbital characteristics, electrostatic properties, and solvent-dependent electronic descriptors of acteoside. The initial three-dimensional structure was obtained from the PubChem database (CID 5281800). Because of the high conformational flexibility of acteoside, a conformational search was conducted before the DFT calculations.

A total of 300 conformers were generated using the ETKDGv3 algorithm implemented in RDKit and preoptimized with the MMFF94s force field. Structures with relative energies greater than 6.0 kcal mol^–1^ were excluded, and conformational redundancy was reduced using an RMSD threshold of 0.5 Å. Among the retained conformers, the structure with the lowest MMFF94s energy was selected for quantum-chemical optimization.

The selected conformer was optimized in the gas phase by using the B3LYP functional and the 6-311G­(d,p) basis set. This level of theory has previously been applied to phenylpropanoid glycosides, caffeoyl-containing natural products, and related polyhydroxylated phenolic systems, providing a computationally tractable description of their molecular geometries, vibrational properties, and electronic characteristics.^34–37^ The polarization functions included in the basis set are particularly relevant to the description of the numerous oxygen-containing functional groups and intramolecular hydrogen-bonding contacts present in acteoside.

The resulting gas-phase geometry was subsequently used as the starting structure for separate reoptimizations in water and DMSO by using the SMD implicit solvation model. Water was used to represent an implicit aqueous medium, whereas DMSO was included, because it was used in the preparation of the experimental stock solution. No independent conformational search was performed in either of the solvents. No geometric constraints were applied during any optimization, and the neutral singlet state was used for all calculations. Harmonic vibrational frequency calculations confirmed the absence of imaginary frequencies for all three structures, identifying them as local minima on their respective potential energy surfaces. These structures are not necessarily the global minima or dominant conformations in each environment.

All quantum-chemical calculations were carried out using Gaussian 16.^38^ The optimized geometries, highest occupied molecular orbital, lowest unoccupied molecular orbital, electron-density distributions, and molecular electrostatic potential (MEP) maps were visualized using GaussView 6.0.^39^ The electronic descriptors reported in this study were obtained from the fully optimized and frequency-confirmed geometries.

Within a Koopmans-type approximation applied to the Kohn–Sham orbital energies, the ionization energy and electron affinity were estimated as

I=−E_HOMOandA=−E_LUMO

The electronegativity, electronic chemical potential, chemical hardness, global softness, and electrophilicity index were calculated according to

χ=(I+A)/2

μ=−χ

η=(I−A)/2

S=1/(2η)

and

$$\omega =\mu^{2}/\left(2\eta \right)$$

The gas-phase calculations describe the intrinsic properties of isolated acteoside, whereas the solution-phase calculations account for the effects of continuum solvation on its geometry and electronic structure.

### Molecular Electrostatic Potential (MEP)

The molecular electrostatic potential surfaces were generated on the electron-density isosurface defined by an isovalue of 0.001 au. To enable direct comparison among the gas-phase, water, and DMSO calculations, the same electrostatic-potential color scale, ranging from −0.140 to +0.140 au, was applied to all three surfaces. The MEP surfaces were visualized using GaussView 6.0.

On the resulting surfaces, red regions correspond to the most negative electrostatic potential and identify electron-rich regions that are more favorable for the interaction with electrophilic species. Blue regions correspond to the most positive electrostatic potential and indicate electron-poor regions that are more favorable for the interaction with nucleophilic species. Intermediate colors represent regions with an electrostatic potential between these limits.

The most negative electrostatic potential regions are localized predominantly around the ester carbonyl, glycosidic ether, and selected phenolic or hydroxyl oxygen atoms. Among these sites, the carbonyl and ether oxygen atoms represent the most plausible hydrogen-bond acceptor regions, whereas the hydroxyl groups may participate primarily as hydrogen-bond donors and, depending on their local environment, as comparatively weaker acceptors.^40^

### Noncovalent Interaction Analysis (NCI)

In this study, a comprehensive noncovalent interaction (NCI) analysis was performed to investigate the weak intramolecular interactions governing the structural stability and chemical behavior of acteoside. The NCI analysis was carried out using Multiwfn 3.8 based on the wave function obtained from the optimized geometry calculated at the B3LYP/6-311G­(d,p) level of theory.^40^

Within the NCI framework, the reduced density gradient (RDG) was calculated as a function of the electron density (ρ) and the sign of the second eigenvalue of the electron-density Hessian matrix, sign­(λ_2_)­ρ. This approach enables the identification and visualization of weak noncovalent interactions, including hydrogen bonding, van der Waals interactions, and steric repulsion. RDG isosurfaces were generated to distinguish attractive interactions (negative sign­(λ_2_)­ρ values), weak dispersive van der Waals contacts (sign­(λ_2_)­ρ values close to zero), and repulsive steric interactions (positive sign­(λ_2_)­ρ values).

The resulting grid and isosurface data were imported into Visual Molecular Dynamics (VMD) for three-dimensional visualization.^41^

The NCI surfaces were color-coded according to the sign­(λ_2_)­ρ values, where blue regions represent strong attractive interactions such as hydrogen bonds, green regions correspond to weak van der Waals interactions, and red regions indicate steric repulsion. Furthermore, RDG versus sign­(λ_2_)­ρ scatter plots were generated using GNUplot 6 to provide a quantitative representation of the distribution and strength of the noncovalent interactions.

This NCI analysis provides valuable insights into the weak interaction network of acteoside, contributing to a better understanding of its structural stability, intermolecular interaction characteristics, and potential molecular recognition behavior.^40,41^

### Electron Localization and Topological Mapping Analysis

Electron localization analyses, including the Electron Localization Function (ELF), Localized Orbital Locator (LOL), and electrostatic potential (ESP) surface mapping, were performed using the wave function files generated from the optimized DFT geometry. All calculations were carried out at the B3LYP/6-311G­(d,p) level of theory using Gaussian 16. The resulting wave function (*.wfn) files were imported into Multiwfn 3.8, where ELF and LOL values were computed on a three-dimensional grid covering the molecular space. Two-dimensional contour maps and three-dimensional isosurface visualizations were generated to characterize electron pairing, bonding domains, and orbital localization. Electrostatic potential (ESP) surfaces were calculated on the molecular electron-density isosurface (ρ = 0.001 au) to identify regions of charge accumulation and depletion. All graphical representations, including ELF/LOL surface meshes and ESP projections, were rendered using Multiwfn 3.8 together with GNUplot. These analyses provide detailed insights into the real-space electronic structure, bonding patterns, and reactive sites of acteoside.^42,43^

## Results and Discussion

### Anticancer Activity

The 72-h application of acteoside in the range of 32–2048 μM resulted in a dose-dependent decrease in viability in all cell lines. The IC_50_ values obtained from the 4PL dose–response curves fitted with log_10_-transformed data were determined as follows: MiaPaCa-2: 166.9 μM, HL-60: 214.5 μM, A549: 353.6 μM, and MDA-MB-231: 374.3 μM (Figure 1). Two-way ANOVA performed on the raw viability data showed a significant effect of both the cell line (*p* < 0.0001) and concentration factors (*p* < 0.0001), as well as a significant interaction between the two variables (*p* < 0.0001). This finding confirms that the cell lines exhibit different dose–response profiles to acteoside. The Extra–Sum-of-Squares *F* test applied to compare the IC_50_ values between the cell lines showed that MiaPaCa-2 cells had a significantly lower IC_50_ compared to HL-60, A549, and MDA-MB-231 (*p* < 0.001). The IC_50_ value of HL-60 was also significantly lower than those of A549 and MDA-MB-231 (*p* < 0.001). In contrast, the IC_50_ difference between A549 and MDA-MB-231 was not statistically significant (p = 0.254). These results indicate that the cytotoxic effects of acteoside differ among the studied cell lines, with MiaPaCa-2 cells showing relatively higher sensitivity compared with the other cell lines.

**Figure 1:**
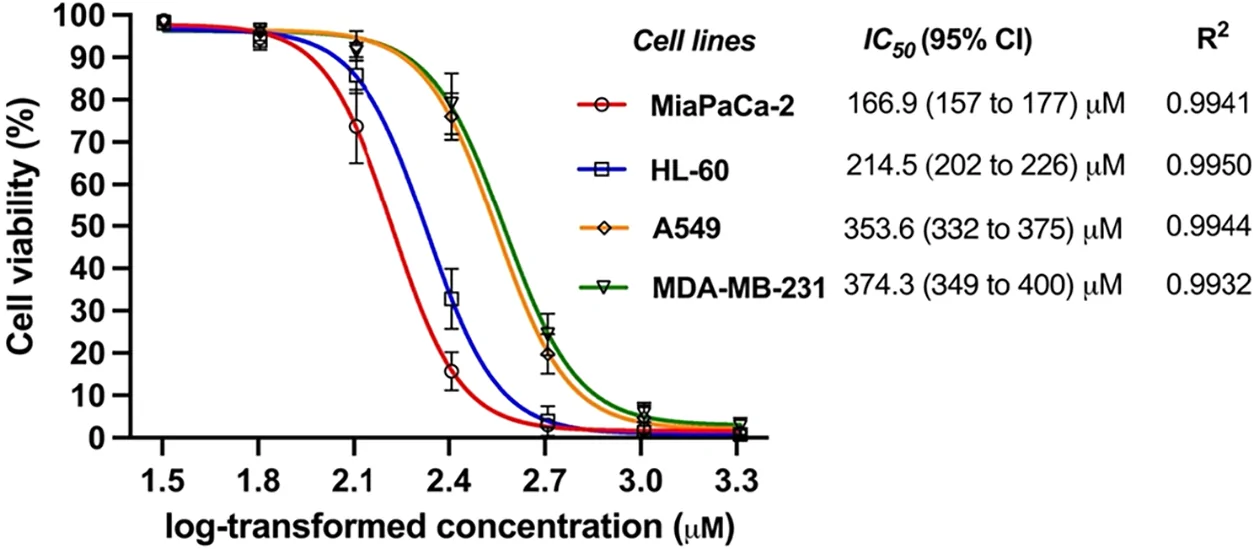
Dose–response curves showing cell viability in MiaPaCa-2, HL-60, A549, and MDA-MB-231 cells against logarithmically transformed concentrations of acteoside. Data represent the mean ± SD of three technical replicates, and the curves were fitted using the four-parameter logistic model.

Among the tested cell lines, MiaPaCa-2 showed the greatest sensitivity to acteoside. This finding may be considered in the context of biological processes previously associated with acteoside and other natural products. Marčetić et al.^21^ described the multifaceted biological properties of acteoside, including cytotoxic, anti-inflammatory, and immunomodulatory activities, while Park^44^ highlighted the potential influence of natural products on metabolic vulnerabilities associated with pancreatic cancer. Although these mechanisms were not directly examined in the present study, they provide a relevant biological context for the comparatively greater sensitivity observed in MiaPaCa-2 cells.

Acteoside has also been associated with the modulation of inflammatory and oxidative-stress pathways. Seo et al.^45^ reported the suppression of HMGB1 release and modulation of the HO-1/Nrf2 axis, whereas Zhu et al.^22^ demonstrated effects on oxidative stress and mitochondrial dynamics. Although these findings were obtained in noncancer experimental models and therefore cannot be directly extrapolated to MiaPaCa-2 cells, they support the broader capacity of acteoside to modulate cellular stress-related processes. Such mechanisms may contribute to differences in cellular sensitivity; however, their involvement in the cytotoxic responses observed in the present study remains to be experimentally established.

In MCF-7 breast cancer cells, acteoside has been reported to interact with estrogen receptor α and estrogen receptor β, functioning as either an estrogen agonist or antagonist.^19,46^ Acteoside, present in the traditional Chinese herbal formula Si-Wu-Tang, has been reported to exhibit significant antiproliferative effects against breast cancer cells.^47^ Acteoside isolated from the plant *Phlomis nissolii* has been reported to show IC_50_ values in breast cancer cell lines, with a concentration of 100 μg/mL exerting significant cytotoxic effects in MCF-7 and MDA-MB-231 cell lines.^48^ In another study, acteoside was reported to exhibit antiproliferative effects in 4T1 breast cancer cells (IC_50_ value of 117 μg/mL).^49^ Within the scope of this study, MDA-MB-231 exhibited the highest IC_50_ value among the tested cell lines, indicating comparatively lower sensitivity to acteoside under the present experimental conditions. This observation may be considered in the context of the distinct biological characteristics of triple-negative breast cancer cells. However, the molecular mechanisms underlying this comparatively lower sensitivity were not directly investigated in the present study and therefore cannot be established from the current data.

Lee et al.^23^ reported that acteoside suppressed HL-60 human promyelocytic leukemia cell proliferation in a time- and concentration-dependent manner, with an IC_50_ of approximately 30 μg/mL. Consistent with the involvement of apoptotic mechanisms in leukemia models, Ma et al.^50^ reported the enhanced apoptosis and caspase activation using an acteoside-loaded formulation, while Akgun-Cagliyan et al.^51^ demonstrated the inhibition of cell growth and induction of apoptosis in K562 and resistant K562 (R-K562) leukemia cells. In the present study, acteoside also produced a cytotoxic response in HL-60 cells, supporting its previously reported antiproliferative activity in leukemia models. However, the underlying apoptotic or cell-cycle mechanisms were not directly investigated in the present study.

Antitumor activity of acteoside has previously been reported in A549 lung cancer cells.^52^ In addition, an acteoside-enriched extract of *Clerodendrum chinense* was reported to exert dose- and time-dependent antiproliferative effects in A549 cells, with an IC_50_ value of 107.08 μg/mL at 72 h.^53^ These previous findings are consistent with the cytotoxic response to acteoside observed in A549 cells in the present study, although direct quantitative comparison should be interpreted cautiously because of differences in the tested preparations and experimental conditions.

Overall, the IC_50_ values obtained in this study demonstrate that the magnitude of the cytotoxic response to acteoside varies among the tested cancer cells. The available literature suggests that such variability may be associated with differences in cellular processes, including oxidative stress, inflammatory signaling, mitochondrial function, and apoptosis. Further studies incorporating gene expression analyses, ROS measurements, mitochondrial membrane potential assessments, and caspase activity would be valuable for elucidating the molecular basis of these differential responses. Taken together, the present findings support the cytotoxic potential of acteoside while highlighting differences in sensitivity among the evaluated cancer cell models.

### Molecular Docking Results and Comparative Binding Analysis

The binding energies and ligand efficiency–based metrics obtained from the molecular docking analyses provide comprehensive insights into the interaction profiles of acteoside and the native inhibitor TAK-285 within the EGFR/HER2 tyrosine kinase domain (Table 1).

**Table 1:** Binding Affinity and Ligand Efficiency Comparison between Acteoside and the Native Reference Inhibitor (TAK-285)^a^

	binding energy (kcal/mol)	LE	FQ	BEI
acteoside	–8.9	0.202	0.777	0.014
TAK-285 (native)	–10.6	0.279	0.948	0.019

a BEI, binding efficiency index; FQ, fit quality; LE, ligand efficiency.

The calculated binding energy for acteoside (−8.9 kcal/mol) indicates a meaningful affinity toward the ATP-binding pocket. Furthermore, its Ligand Efficiency (LE = 0.202), normalized by the number of heavy atoms, together with its Fit Quality (FQ = 0.777), which accounts for the molecular size and structural complexity, demonstrates that the compound accommodates the binding cleft effectively. In addition, the Binding Efficiency Index (BEI = 0.014) reflects a balanced distribution of binding performance relative to the molecular weight of acteoside. For the reference inhibitor TAK-285 extracted from the crystal structure, the predicted binding energy of −10.6 kcal/mol denotes a strong interaction potential with the receptor. The ligand’s LE (0.279) and FQ (0.948) values highlight an efficient and pharmacophorically concentrated interaction pattern within the active site. Moreover, the BEI value (0.019) indicates an effective proportionality between binding energy and molecular mass (Figure 3). Collectively, these docking-derived parameters provide a quantitative basis for understanding how both ligands engage with the catalytic domain of EGFR/HER2.

Preliminary data obtained from molecular docking studies were evaluated to investigate the interaction of acteoside with the epidermal growth factor receptor (3POZ). The calculated binding energy for acteoside was −8.9 kcal/mol. Compared with the reference EGFR/HER2 kinase inhibitor TAK-285, which exhibited a binding energy of −10.6 kcal/mol, the less favorable docking score of acteoside suggests a comparatively weaker predicted interaction with the 3POZ binding site. However, docking-derived binding energies represent computational estimates of ligand–receptor interactions and should not be interpreted as direct measures of inhibitory activity.

In the molecular docking study conducted by Oyeneyin et al.,^25^ the calculated binding energies of the lead compounds (22 and 23), which are 2-alkoxy carbonyl allyl esters targeting the epidermal growth factor receptor (EGFR) in the pancreatic cancer (MiaPaCa-2) cell line, were reported as −6.327 and −7.232 kcal/mol, respectively. These values were reported to exhibit better inhibition at the 3POZ binding site than the standard drug chlorambucil (−6.0 kcal/mol). Similarly, in the present study, although acteoside exhibited a lower binding affinity than the reference EGFR/HER2 kinase inhibitor TAK-285, it demonstrated a strong binding affinity toward the target protein with a binding energy of −8.9 kcal/mol. Furthermore, the lower binding energy of acteoside compared with those reported for the lead compounds (22 and 23) by Oyeneyin et al.^25^ suggests that acteoside may possess a higher binding affinity for the 3POZ binding site of EGFR and could be considered a potential EGFR inhibitor.

In another study, molecular docking studies of the epidermal growth factor receptor with zerumbone and curcumin reported binding energies of −8.0 and −7.6 kcal/mol, respectively, indicating higher binding compared to erlotinib (−7.3 kcal/mol).^54,55^ Within the scope of this study, acteoside exhibited a high binding affinity for the epidermal growth factor receptor, similar to EGFR/HER2 Kinase Inhibitor TAK-285, suggesting that it could be a candidate EGFR inhibitor. Consequently, acteoside may have inhibitory activity potential against EGFR-TK.

Figure 2 presents a comparative interaction profile of acteoside and the reference ligand TAK-285 within the EGFR/HER2 active pocket. Acteoside exhibits an extensive network of conventional hydrogen bonds involving LYS745, VAL726, LEU788, ARG841, ASN842, GLY724, and ASP837, with interaction distances ranging from 1.36 to 2.76 Å. C–H···O interactions are also observed, together with a π-cation interaction involving LYS745. In addition, hydrophobic contacts, including alkyl and π-alkyl interactions, are observed with ALA722, PHE723, ALA743, VAL726, and LYS745, with distances extending to approximately 5.42 Å. In comparison, TAK-285 displays a more diverse interaction profile. The reference ligand forms a conventional hydrogen bond with THR790 and a C–H···O interaction with LYS745, while additional interactions include halogen contacts involving ASN842 and GLY724, π-cation and π-anion interactions with LYS745 and ASP855, respectively, and a π-donor hydrogen bond with ASP855. π-sigma and π–π T-shaped interactions are also observed, together with alkyl and π-alkyl contacts involving ALA743, VAL726, LEU792, MET766, LEU788, and LEU858. Overall, the interaction profile indicates that acteoside is characterized by an extensive hydrogen-bonding network accompanied by hydrophobic interactions, whereas TAK-285 engages the binding pocket through a broader range of hydrogen-bonding, hydrophobic, aromatic, electrostatic, and halogen-mediated interactions.

**Figure 2:**
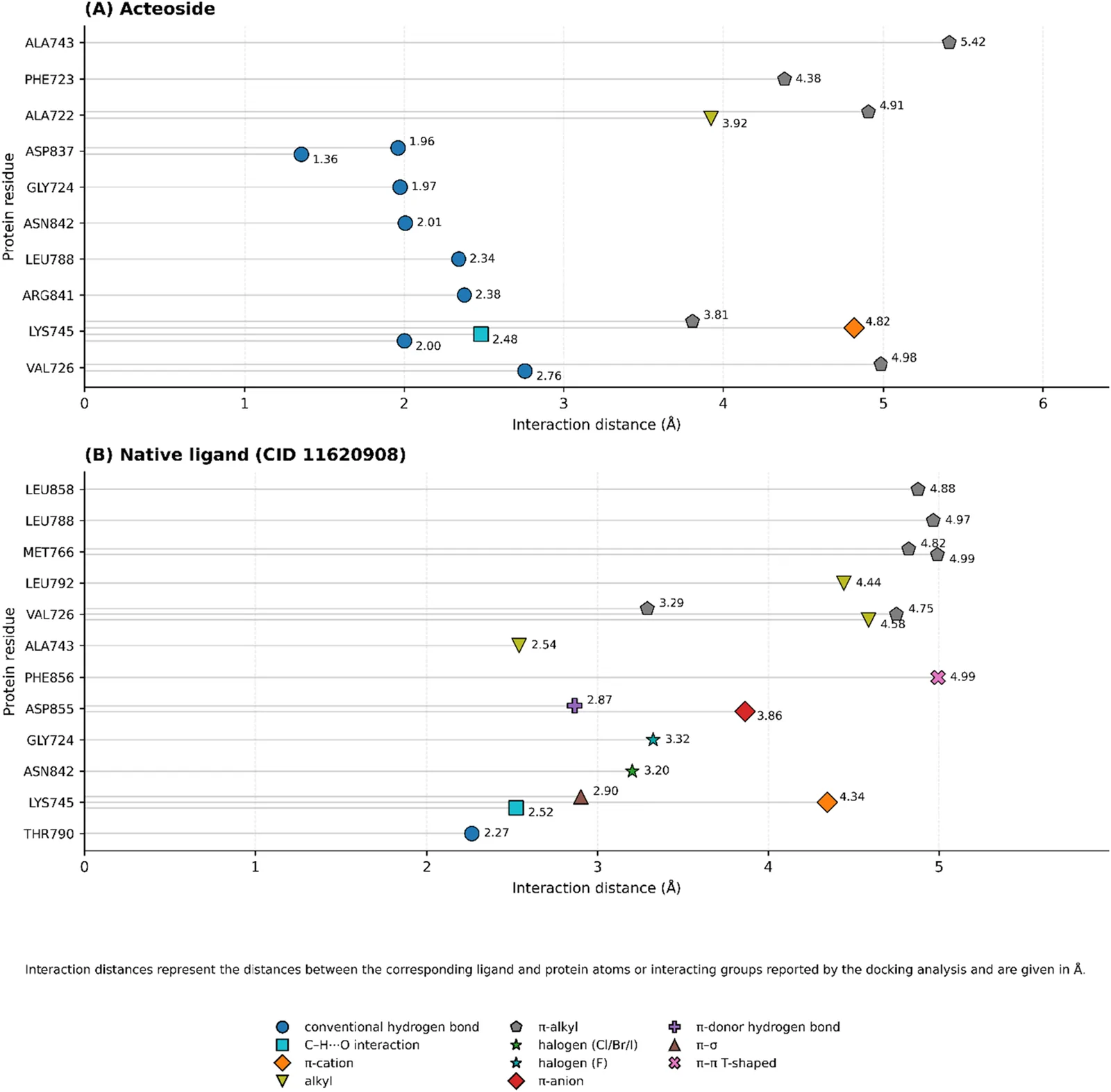
Interaction profile of key amino acid residues involved in the binding of (A) acteoside and (B) TAK-285 (CID 11620908) within the EGFR/HER2 active pocket. Different symbols represent distinct interaction types, and the corresponding interaction distances are given in Å.

The three-dimensional binding pose and two-dimensional interaction profile of acteoside (Figure 3) reveal that the ligand occupies the ATP-binding pocket through an extensive network of hydrogen bonds. Conventional hydrogen bonds are formed with VAL726:HN, LYS745:HZ1, ARG841:HH22, LEU788:O, ASN842:OD1, GLY724:O, and ASP837:OD1, with interaction distances ranging from 1.36 to 2.76 Å. A C–H···O interaction involving LYS745:HE2, with an interaction distance of 2.48 Å, further contributes to the ligand–protein interaction network. Moreover, hydrophobic alkyl and π-alkyl interactions with ALA722, PHE723, ALA743, VAL726, and LYS745 provide additional contacts within the ATP-binding cleft. Collectively, these interactions indicate that acteoside is characterized by an extensive hydrogen-bonding network accompanied by complementary hydrophobic interactions.

**Figure 3:**
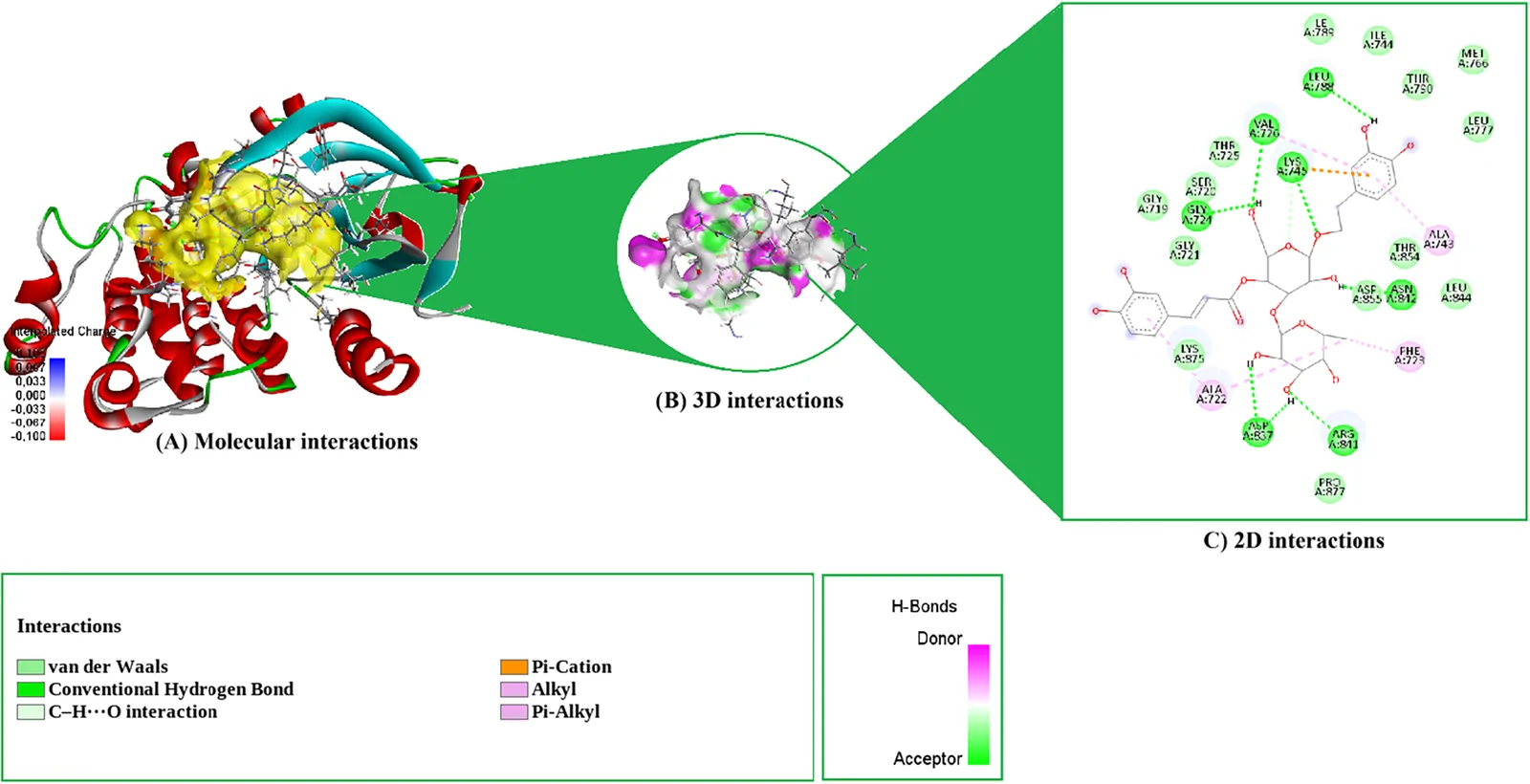
Molecular docking analysis of acteoside within the EGFR/HER2 tyrosine kinase domain. (A) Overall binding orientation of acteoside within the ATP-binding pocket of EGFR/HER2, with the yellow surface representing the molecular surface of the bound ligand (B) Three-dimensional protein–ligand interaction profile highlighting the binding environment and hydrogen-bond donor/acceptor regions. (C) Two-dimensional interaction map illustrating the amino acid residues and intermolecular interactions involved in ligand recognition and binding stabilization.

The docking pose and interaction map of the reference ligand TAK-285 (Figure 4) demonstrate a diverse interaction pattern within the EGFR/HER2 catalytic pocket. The ligand forms halogen interactions involving ASN842:OD1 and GLY724:O, with interaction distances of 3.20 and 3.32 Å, respectively. Electrostatic interactions include a π-cation interaction with LYS745:NZ at 4.34 Å and a π-anion interaction with ASP855:OD1 at 3.86 Å. A π-donor hydrogen bond involving ASP855:HN and a π-sigma interaction involving LYS745:HD2 further contribute to the interaction network, with reported distances of 2.87 and 2.90 Å, respectively. In addition, a π–π T-shaped interaction with PHE856 is observed at 4.99 Å. Multiple alkyl and π-alkyl interactions involving ALA743, VAL726, LEU792, MET766, LEU788, and LEU858 provide additional hydrophobic contacts within the binding pocket. Overall, TAK-285 exhibits a heterogeneous interaction profile comprising hydrogen-bonding, hydrophobic, aromatic, electrostatic, and halogen-mediated interactions, in contrast to the more hydrogen-bond-rich interaction pattern observed for acteoside.

**Figure 4:**
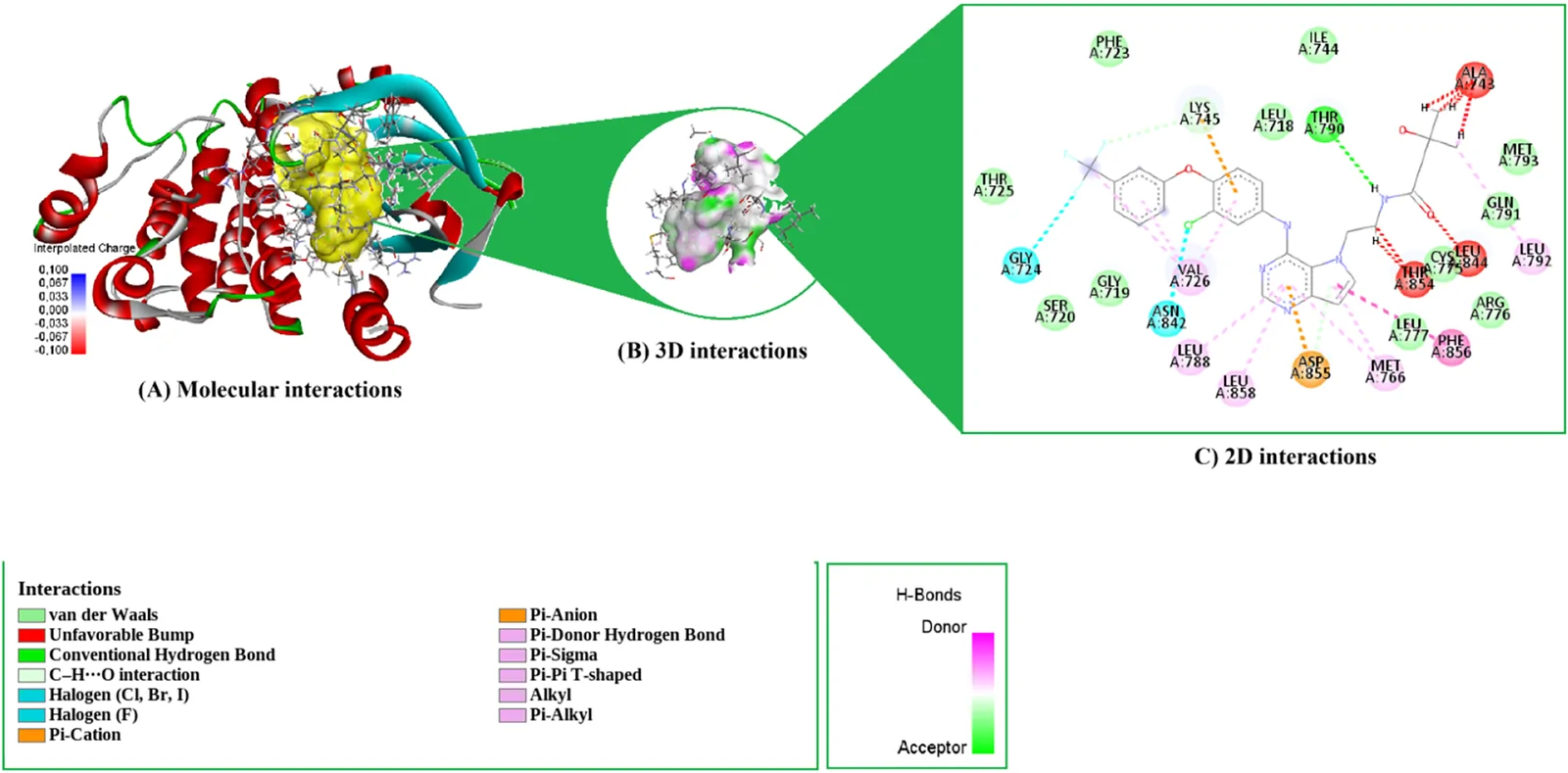
Molecular docking analysis of TAK-285 within the EGFR/HER2 tyrosine kinase domain. (A) Overall binding orientation of TAK-285 within the ATP-binding pocket of EGFR/HER2, with the yellow surface representing the molecular surface of the bound ligand (B) Three-dimensional protein–ligand interaction profile highlighting the binding environment and hydrogen-bond donor/acceptor regions. (C) Two-dimensional interaction map illustrating the amino acid residues and intermolecular interactions responsible for ligand recognition and binding stabilization.

### Frontier Molecular Orbitals and Global Reactivity Descriptors

Frontier molecular orbital analysis was performed to examine the solvent-dependent electronic characteristics of the acteoside. The corresponding orbital energies and global reactivity descriptors are summarized in Table 2.

**Table 2:** Frontier Molecular Orbital Energies and Global Reactivity Descriptors of Acteoside Obtained from DFT Calculations^a^

parameter	gas	water	DMSO
*E*_HOMO (eV)	–5.148	–5.714	–5.574
*E*_LUMO (eV)	–2.259	–2.060	–2.022
Δ*E*_gap (eV)	2.889	3.654	3.552
*I* (eV)	5.148	5.714	5.574
*A* (eV)	2.259	2.060	2.022
χ (eV)	3.704	3.887	3.798
μ (eV)	–3.704	–3.887	–3.798
η (eV)	1.445	1.827	1.776
*S* (eV^–1^)	0.692	0.547	0.563
ω (eV)	4.748	4.135	4.060
dipole (D)	8.095	9.677	8.811

a *E*_HOMO, highest occupied molecular orbital energy; *E*_LUMO, lowest unoccupied molecular orbital energy; Δ*E*_gap, HOMO–LUMO energy gap; *I*, ionization energy; *A*, electron affinity; χ, electronegativity; μ, electronic chemical potential; η, chemical hardness; *S*, global softness; ω, electrophilicity index; Dipole, dipole moment.

In the gas phase, acteoside exhibited HOMO and LUMO energies of −5.148 and −2.259 eV, respectively, corresponding to a HOMO–LUMO energy gap of 2.889 eV. Reoptimization in the SMD water and DMSO environments resulted in energy gaps of 3.654 and 3.552 eV, respectively. Additional computational data are provided in Supporting Table S1. No major conformational rearrangement was observed upon reoptimization in water or DMSO, and the overall molecular geometry remained similar to that obtained in the gas phase, indicating relatively limited solvent-induced structural relaxation within the SMD model. Thus, the frontier-orbital gap increased in both SMD-polarized electrostatic fields relative to the gas phase. Comparable levels of theory have previously been applied to phenylpropanoid glycosides to evaluate their electron-donating and electron-accepting capacity, and the present values fall within the moderate-reactivity range described for organic molecules computed at the same level of theory (e.g., a gap of ∼3.2 eV associated with low intrinsic reactivity). The HOMO and LUMO distributions shown in Figure 5 correspond to the gas-phase optimized structure and provide a representative spatial description of acteoside’s frontier orbitals. The corresponding HOMO and LUMO distributions obtained in water and DMSO are provided in Figure S1. The HOMO density is predominantly localized over one aromatic/conjugated region, whereas the LUMO density is mainly distributed over a different aromatic/conjugated region. This spatial separation indicates that the electron-donor and electron-acceptor characteristics are distributed over distinct molecular regions. Such spatial information cannot be obtained from the numerical HOMO–LUMO gap or from the optimized geometry alone. The orbital maps therefore complement the global energy descriptors by identifying regions that may participate in donor–acceptor and polar interactions. Comparison of the optimized wave functions (Figures 5 and S1) indicates that the qualitative HOMO and LUMO localization remains similar in the gas phase, water, and DMSO calculations. This preservation is expected for a structurally stable, closed-shell molecule such as acteoside: the SMD model modifies the orbital energies and molecular polarization through a polarized electrostatic field, but it does not introduce explicit solvent molecules or new chemical bonds. A pronounced change in the HOMO/LUMO character would instead suggest an unusual gas-phase electronic instability, which is not expected for this molecular system. Accordingly, the solvent-dependent electronic effects are expressed primarily through changes in the orbital energy levels, energy gaps, and other global descriptors, while the qualitative orbital character remains preserved.

**Figure 5:**
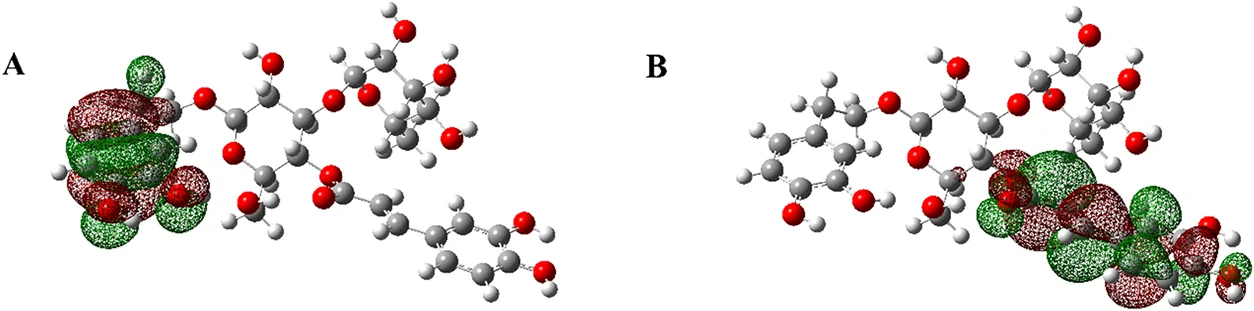
Frontier molecular orbital distributions of acteoside calculated for the gas-phase optimized structure at the B3LYP/6-311G­(d,p) level: (A) highest occupied molecular orbital (HOMO) and (B) lowest unoccupied molecular orbital (LUMO).

Using this definition, the gas-phase hardness and softness values were 1.445 and 0.692 eV^–1^, respectively. In water, the corresponding values were 1.827 and 0.547 eV^–1^, whereas in DMSO, they were 1.776 and 0.282 eV^–1^. The increase in hardness and corresponding decrease in softness indicate reduced frontier-orbital-based electronic deformability in the implicit solvent environments.

The electrophilicity index decreased from 4.748 eV in the gas phase to 4.135 eV in water and 4.060 eV in DMSO. Within the adopted descriptor framework, this change indicates a modest reduction in global electrophilicity in the SMD-polarized electrostatic fields. The electronegativity and chemical potential varied less markedly, suggesting that continuum solvation had a stronger effect on the HOMO–LUMO separation than on the midpoint of the frontier-orbital energies.

The calculated dipole moment increased from 8.095 D in the gas phase to 9.677 D in water and 8.811 D in DMSO, indicating enhanced molecular polarization upon implicit solvation. Notably, the solvent-dependent changes in the calculated descriptors do not follow a single monotonic gas–water–DMSO sequence. This behavior reflects descriptor-specific responses to the combined effects of continuum solvation and solvent-dependent geometric relaxation rather than a simple dependence on solvent polarity. Within the present SMD calculations, water produced the largest increase in both dipole moment and HOMO–LUMO gap, whereas the electrophilicity index was slightly lower in DMSO than in water. Accordingly, the water–DMSO differences should be interpreted as distinct solvent-dependent responses of the optimized electronic structure rather than as a uniform polarity-driven trend.

These results describe relative solvent-dependent changes in the electronic structure of acteoside within the selected computational model and should not be interpreted as direct measures of thermodynamic stability, electron transfer rates, polarizability, or biological activity, which depend on additional structural, environmental, and kinetic factors. Nevertheless, the DFT results provide complementary molecular level information for interpreting the docking results. The increased dipole moment in the SMD-polarized electrostatic fields, together with the spatially differentiated HOMO and LUMO regions shown in Figure 5, indicates that acteoside has electronically distinct molecular regions that may contribute to polar and hydrogen-bond-mediated interactions within the EGFR/HER2 binding pocket. This interpretation is consistent with the docking analysis in which acteoside exhibited an extensive network of conventional hydrogen bonds involving VAL726, LYS745, ARG841, LEU788, ASN842, GLY724, and ASP837, together with complementary hydrophobic interactions. The preservation of the qualitative frontier-orbital localization upon inclusion of the SMD fields is an expected result for a structurally stable closed-shell molecule and does not imply that the orbital descriptors directly determine protein binding. The DFT descriptors should therefore be interpreted as an electronic-structure context that complements, rather than replaces, the interaction pattern identified by molecular docking.

### Molecular Electrostatic Potential Surface Analysis

The molecular electrostatic potential maps of acteoside provide a spatial representation of the electrostatic potential over its molecular surface and reveal distinct electron-rich and -poor regions in the gas phase, water, and DMSO (Figure 6). In the adopted color scale, red and orange regions correspond to negative electrostatic potential, whereas cyan and blue regions represent positive electrostatic potentials. The most negative regions are localized primarily around the ester carbonyl and glycosidic ether oxygen atoms together with selected phenolic and aliphatic hydroxyl oxygen atoms. The carbonyl and ether oxygen atoms therefore constitute the most plausible hydrogen-bond acceptor regions, whereas the hydroxyl groups provide complementary donor surfaces through the strongly positive potential concentrated around their hydrogen atoms.

**Figure 6:**
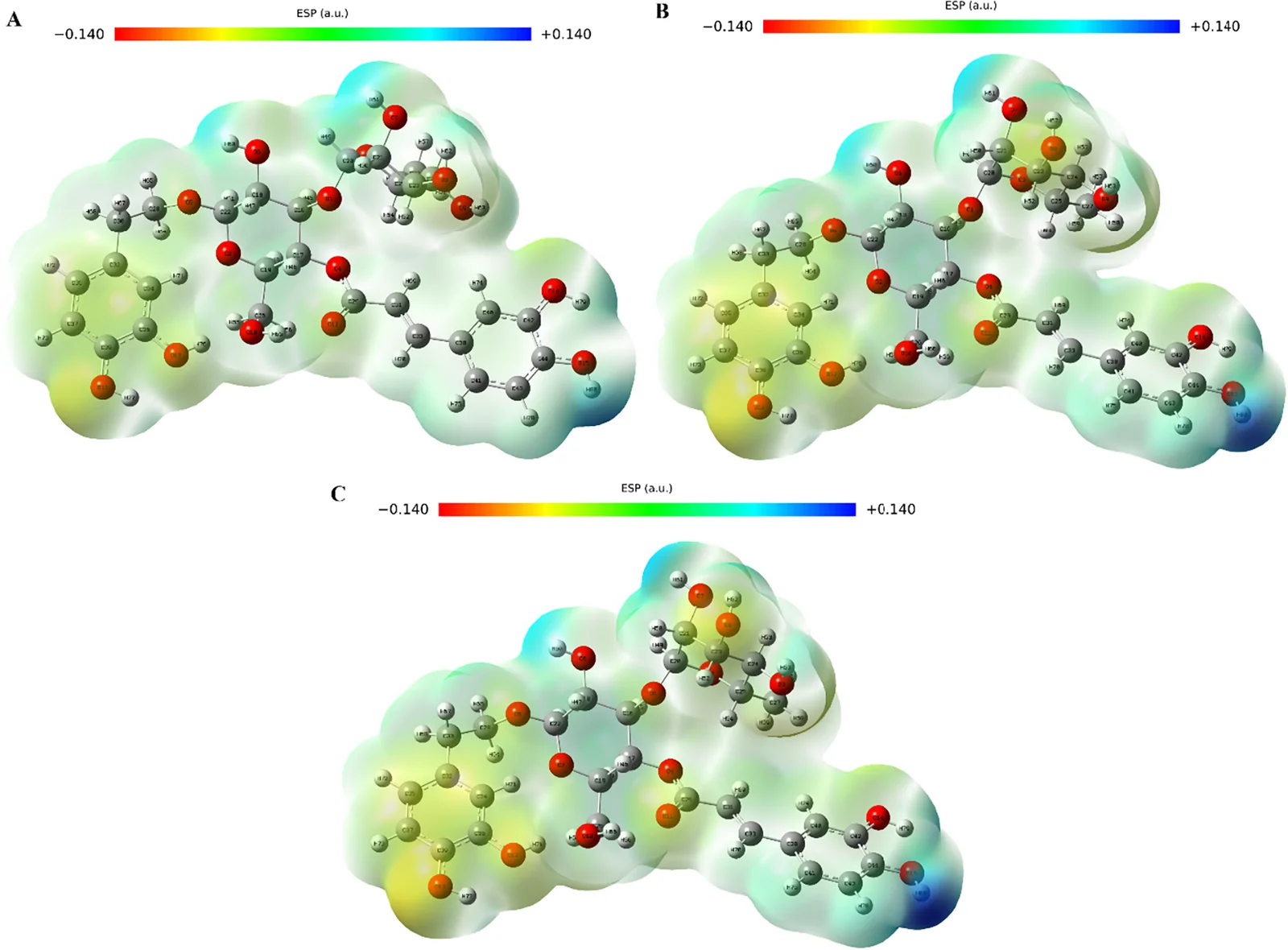
Molecular electrostatic potential (MEP) surfaces of acteoside calculated at the B3LYP/6-311G­(d,p) level. (A) Gas phase. (B) DMSO. (C) Water. Red and orange regions indicate negative electrostatic potential (electron-rich regions), whereas cyan and blue regions represent positive electrostatic potential (electron-deficient regions).

The electrostatic-potential range increases from −0.08329 to +0.08329 au in the gas phase to −0.09841 to +0.09841 au in DMSO and −0.107 to +0.107 au in water. This progressive broadening indicates enhanced polarization of the molecular electrostatic surface in the implicit polar environments, with the largest separation between negative and positive potential regions observed in water. The locations of the principal donor and acceptor regions remain broadly conserved across the three environments, although their spatial extent and magnitude change following solvent-induced geometric relaxation and electronic polarization.

The oxygen-rich negative potential regions and the positive potential hydroxyl hydrogen surfaces are qualitatively consistent with the hydrogen bond rich interaction pattern identified in the docking analysis. In particular, these complementary electrostatic regions may facilitate interactions with appropriately positioned donor and acceptor residues within the EGFR/HER2-binding cavity.

### Noncovalent Interaction Analysis (NCI)

Noncovalent interaction analysis was performed for the gas-, water-, and DMSO-phase optimized geometries of acteoside to examine solvent-dependent changes in its intramolecular interaction pattern (Figure 7). In all three environments, the RDG versus sign (λ_2_) ρplots display negative, near-zero, and positive regions associated with attractive interactions, weak dispersion-dominated contacts, and steric repulsion, respectively. This interpretation follows the established NCI framework based on the reduced density gradient and the sign of the second eigenvalue of the electron-density Hessian.^40,56^

**Figure 7:**
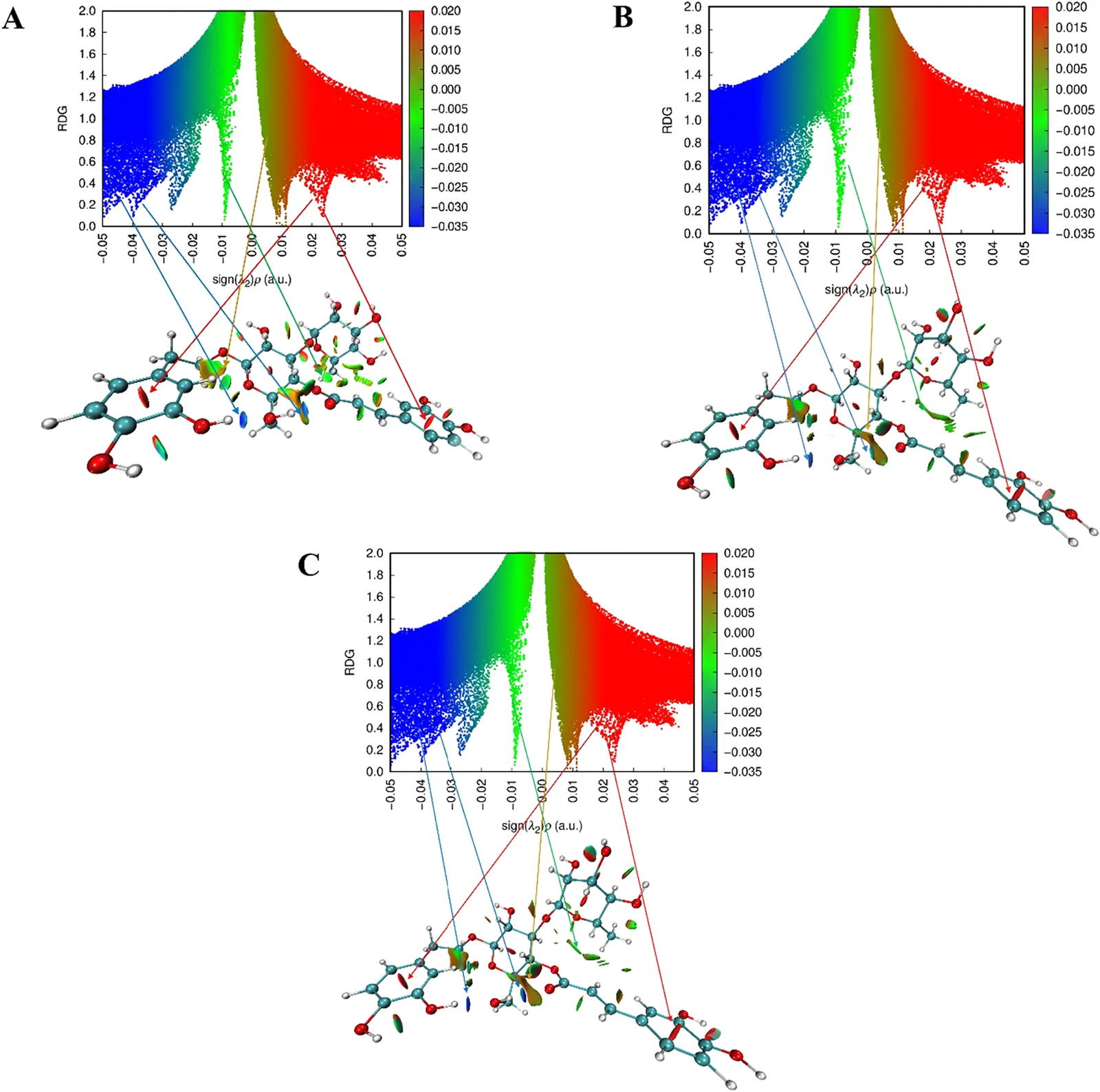
Noncovalent interaction analysis of acteoside in (A) the gas phase, (B) water, and (C) DMSO. The upper panels present plots of the reduced density gradient (RDG) against sign­(λ_2_)­ρ, and the lower panels show the corresponding three-dimensional RDG isosurfaces mapped onto the optimized molecular geometries. Blue and cyan regions represent strongly attractive interactions, green regions indicate weak dispersion-dominated contacts, and red regions denote steric repulsion.

The corresponding three-dimensional isosurfaces reveal localized blue and cyan regions between the spatially adjacent hydroxyl and oxygen-containing groups. On the basis of their spatial positions, these features are assigned primarily to attractive intramolecular contacts involving the oxygen-rich regions of acteoside. More widely distributed green isosurfaces occur throughout the caffeoyl, glycosidic, and aromatic portions of the molecule, indicating that weak dispersion interactions contribute to the conformational organization. Localized red regions are mainly observed within or near-constrained ring environments and are consistent with steric repulsion.

Reoptimization in water and DMSO alters the spatial distribution and extent of the attractive NCI regions relative to those of the gas-phase geometry. These differences reflect solvent-induced conformational relaxation and electronic polarization within the SMD continuum model. However, the NCI surfaces do not provide interaction energies, and differences in their apparent size or number should not be interpreted as direct quantitative measures of hydrogen-bond strength.

The overall profiles obtained in water and DMSO retain the same principal interaction classes observed in the gas phase, although their spatial arrangements change with molecular conformation. The persistence of attractive regions around the hydroxyl- and oxygen-rich portions of the molecule indicates that these functional groups remain available for polar intermolecular interactions. Nevertheless, the present analysis describes only intramolecular interactions within the optimized acteoside structures and does not directly simulate interactions with solvent molecules, amino acid residues, or the protein binding pocket.

Because the SMD model treats the solvent as a polarizable continuum, the observed differences arise from solvent-induced geometric and electronic changes rather than from explicit solute–solvent hydrogen bonding.

### Electron Localization (LOL/ELF) and ESP Analysis

The Electron Localization Function (ELF) and Localized Orbital Locator (LOL) maps provide complementary descriptions of electron localization within acteoside (Figure 8). The maps show variations in localization across the oxygen-containing groups, aromatic moieties, and glycosidic framework. These analyses characterize the electronic structure of the isolated molecule and do not directly quantify ligand–protein interactions. The molecular electrostatic potential is presented separately in Figure 6.

**Figure 8:**
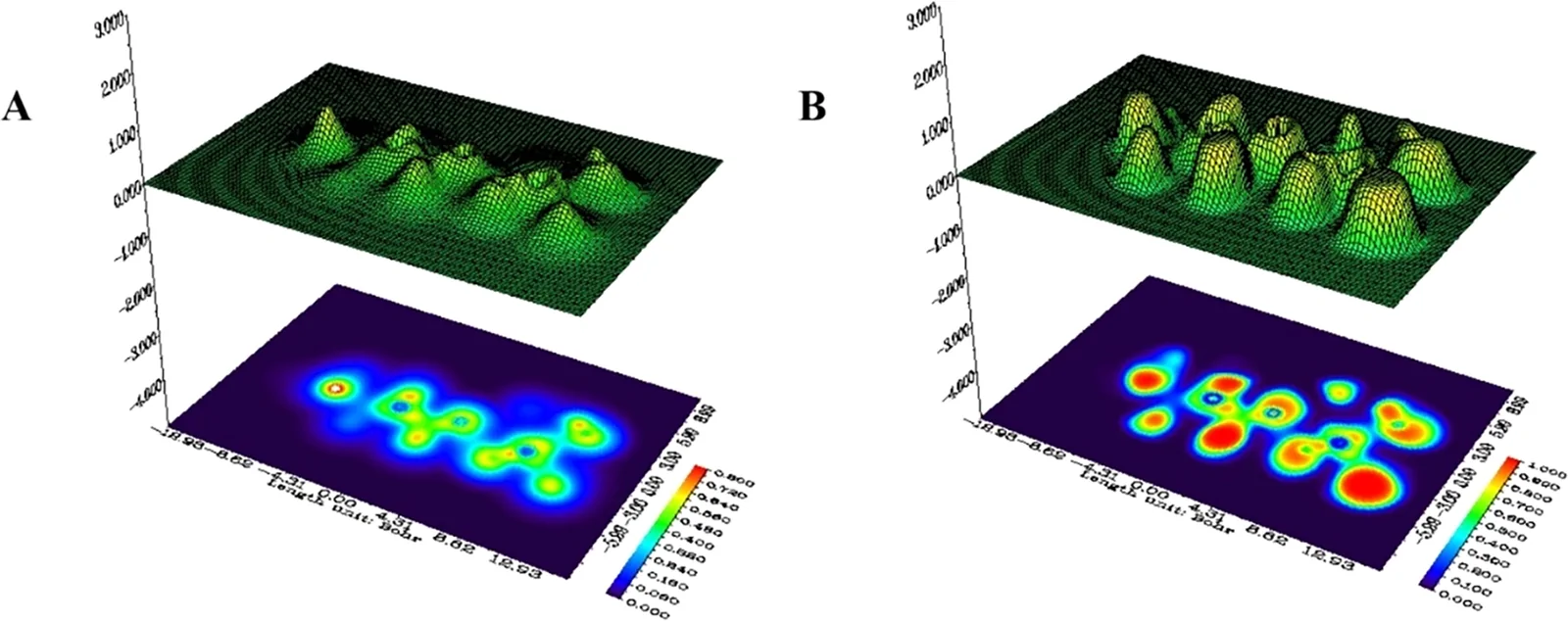
Electron localization maps of acteoside: (A) Localized Orbital Locator (LOL) and (B) Electron Localization Function (ELF). Upper panels show three-dimensional representations, and lower panels show the corresponding two-dimensional color maps. The color scale ranges from 0.00 (blue, low localization) to 1.00 (red, high localization).

The electrostatic potential distribution shown in Figure 8C is consistent with the charge-separation pattern described in Figure 6, while providing a complementary visualization of the molecular electronic surface. Rather than repeating the detailed ESP interpretation, the present analysis emphasizes the relationship between the electrostatic distribution and the localized electron density features identified by the ELF and LOL maps.

Taken together, the ELF and LOL analyses reveal a heterogeneous electronic architecture in acteoside, characterized by localized electron density regions associated with its oxygen-rich and aromatic moieties. These features, together with the electrostatic distribution shown in Figure 8C, provide a complementary electronic basis for the hydrogen-bonding and other noncovalent interactions observed in the docking analysis.

## Conclusion

Acteoside exhibited varying levels of cytotoxicity among the tested cell lines with MiaPaCa-2 pancreatic cancer cells showing the highest sensitivity. Its dose-dependent inhibition of cell viability suggests that it may possess an anticancer potential. The present findings indicate differences in the magnitude of cytotoxic responses among the tested cell lines together with computationally predicted interactions of acteoside with EGFR/HER2. Collectively, these findings provide preliminary evidence supporting further investigation of acteoside in pancreatic cancer. Additional *in vitro* and *in vivo* studies are required to clarify their therapeutic potential and underlying mechanisms of action.

## Supplementary Material





## Data Availability

The data sets generated during and/or analyzed during the current study are available from the corresponding author on reasonable request.

## Notes

Summary of harmonic-frequency verification and corresponding Gaussian output files (Table S1); and frontier molecular orbital (FMO) distributions of acteoside calculated at the B3LYP/6-311G­(d,p) level in water and DMSO (Figure S1) (PDF)
Three Gaussian output files referenced in Table S1 (ZIP)
