# Supplementary material for: A Comprehensive In Vitro and In Silico Study of Acteoside: Cytotoxicity, Molecular Interactions, and DFT Reactivity Profiles
Source: ACS Omega. 2026 Oct 26;11(44):68290–303. doi: 10.1021/acsomega.6c04617 (PMC13756709; doi:10.1021/acsomega.6c04617)
Supplement: Supplementary file 1 [file ao6c04617_si_001.pdf]

# A Comprehensive *in vitro* and *in silico* study of Acteoside: Cytotoxicity, Molecular Interactions, and DFT reactivity profiles

**Erdi Can Aytar[1*], Metin Çalışkan[2], Emine İncilay Torunoğlu[3]**

[1*]Uşak University, Faculty of Agriculture, Department of Horticulture, Uşak, Türkiye
[2]Uşak University, Faculty of Medicine, Department of Medical Biology, Uşak, Türkiye
[3]Necmettin Erbakan University, Faculty of Medicine, Department of Medical Biochemistry, Konya, Türkiye

**Corresponding Author:** Dr. Erdi Can Aytar
erdicanaytar@gmail.com

**Table S1**. Summary of harmonic-frequency verification and corresponding Gaussian output files.

| Environment | Lowest frequency ($cm^{-1}$) | Imaginary frequencies | Output file |
|---|---|---|---|
| **Gas phase** | 10.0021 | 0 | Acteosidegaslog.txt |
| **Water** | 8.1083 | 0 | Acteosidewaterlog.txt |
| **DMSO** | 8.6070 | 0 | Acteosidedmsolog.txt |

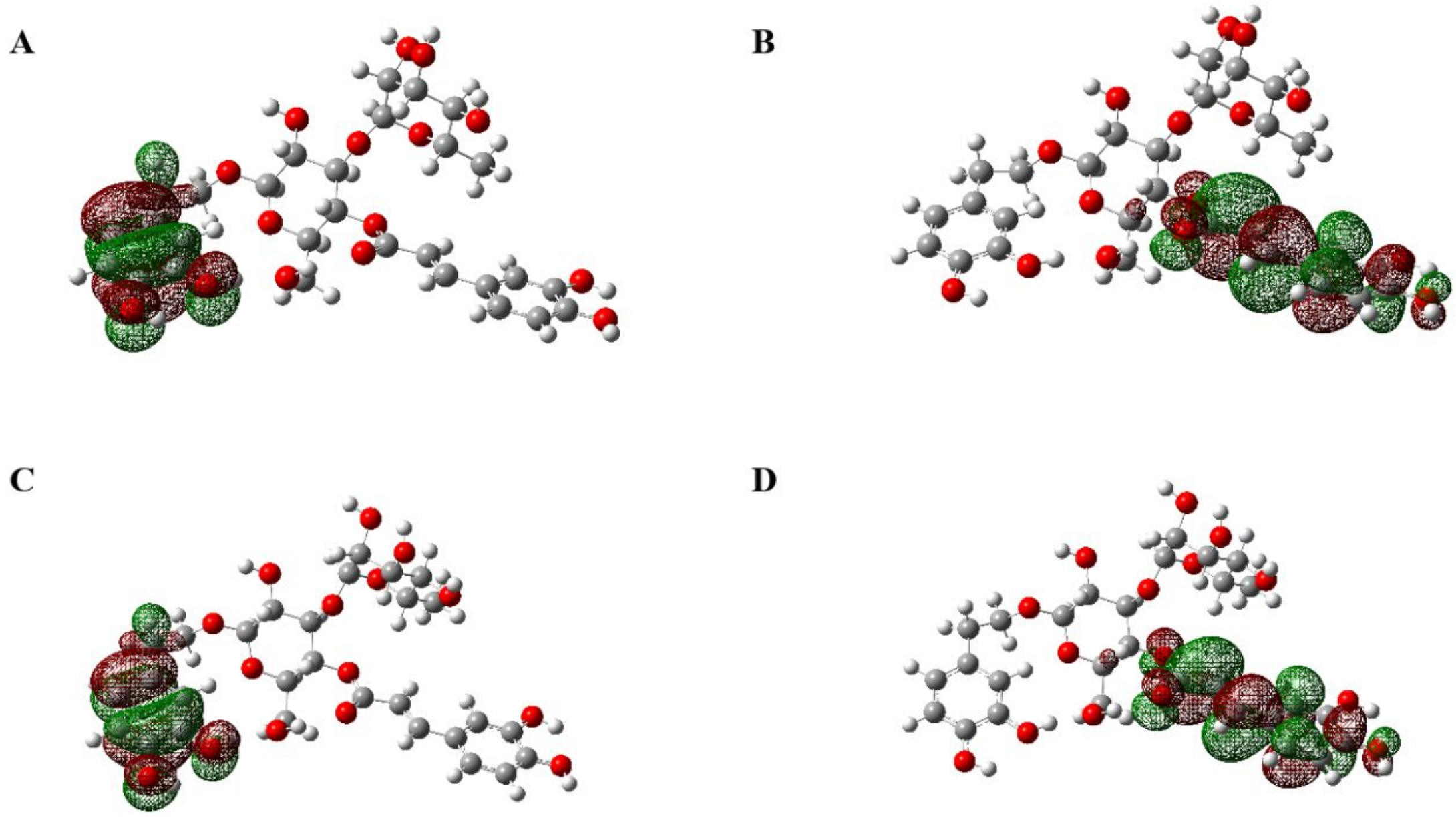


**Figure S1.** Frontier molecular orbital (FMO) distributions of acteoside calculated at the B3LYP/6-311G(d,p) level in water and DMSO: (A) HOMO in water, (B) LUMO in water, (C) HOMO in DMSO, and (D) LUMO in DMSO.
